# Longitudinal qualitative research in medical education

**DOI:** 10.1007/s40037-017-0374-9

**Published:** 2017-08-24

**Authors:** Dorene F. Balmer, Boyd F. Richards

**Affiliations:** 1 Perelman School of Medicine, University of Pennsylvania, Philadelphia, PA USA; 2 University of Utah School of Medicine, Salt Lake City, UT USA

A Qualitative Space highlights research approaches that push readers and scholars deeper into qualitative methods and methodologies. Contributors to *A Qualitative Space *may: advance new ideas about qualitative methodologies, methods, and/or techniques; debate current and historical trends in qualitative research; craft and share nuanced reflections on how data collection methods should be revised or modified; reflect on the epistemological bases of qualitative research; or argue that some qualitative practices should end. Share your thoughts on Twitter using the hashtag: #aqualspace.

‘*Patience is in the living. Time opens out to you.*’ Claudia Rankine [1]

## Introduction

Longitudinal qualitative research (LQR) is an approach to research in which data are collected from the same participants to assess change through time. These serial responses position LQR to bring oft-overlooked, temporal dimensions of phenomena to the fore of inquiry. Nevertheless, the method is infrequently used and not well described in the health professions education literature. In response, we have written this paper. We rely on our experience and review of the literature, particularly Saldaña’s [2] LQR primer, to introduce the fundamentals of LQR (the what, why, who, and how). We share our LQR study as an example and highlight personal reflections about doing LQR. We end with a set of guiding principles for doing LQR that emerged from our reflections.

## What is LQR and why is it used?

At a fundamental level, LQR is characterized by qualitative inquiry that involves a series of data collection with a sample of participants. Data collection is spaced across a period of time that is long enough for researchers to observe, describe, and analyze substantive change in the phenomena under investigation [2–7]. It stands to reason that LQR tends to revolve around issues of time and change, such as how and why an individual student’s feelings and beliefs about their role in the national healthcare system changes throughout their four years of medical school, especially in comparison with group norms. From our perspective, this is the power of LQR: being able to juxtapose an understanding of changes within individuals, with changes in group norms [2, 3]. Similarities and differences in individual stories become evident when those stories are serially collected and analyzed in the shadow of group norms [4, 8].

To be sure, what constitutes ‘enough’ time and what constitutes ‘substantive’ change is relative, making it difficult to discriminate between qualitative research that is or is not LQR. The timeframe between the first and last data collections needed for the LQR ‘label’ to apply is debatable. Some suggest a minimum of nine months [3], others one year [5]; the key is that inquiry lasts long enough and probes deep enough to discern a meaningful change in the phenomena being studied [2, 6].

LQR may be confused with other approaches to qualitative research that have similar types of data collection and analysis, or similar relationships with participants. To avoid confusion, we include, in Table 1, a bulleted comparison across LQR and other approaches along three dimensions: the focus of study, the type of data collection and analysis, and the nature of the research relationship. As displayed in the table, LQR tends to focus on change through time in individuals and in groups rather than on the culture within which the group exists, such as in ethnographic research. It relies on data collection and analysis of both individuals and groups, moving beyond the singular life focus of biography. And LQR tends to foster deep personal understandings between researchers and participants, as individuals and as a group, but without an explicit attempt to empower participants, such as in participatory action research.

**Table 1:** Key features of longitudinal qualitative research and approaches to qualitative research

	Longitudinal qualitative research	Ethnography [15]	Participatory action research [16]	Biography [17]
Focus of study	Exploring temporal dimension of a phenomenon in individuals and in groups	Describing and interpreting a cultural or social group	Seeking collaborative change by actively engaging participants	Exploring the life of an individual
Type of data	Cross sectional and longitudinal data, typically collected via interviews with a relatively small group of individuals	Typically prolonged observation of, and ad hoc field interviews with, members of cultural or social group	Typically cross sectional data collected from multiple individuals	Longitudinal data collected via interviews and/or existing documents and archives
Nature of research relationship	Increasingly personal due to time, repetition, and familiarity	Determined by the stance of the researcher (detached observer to complete participant)	Empowering and collaborative	Singular; Varies from close/personal to more detached

## Who might consider LQR?

To do LQR, researchers should possess knowledge and skills to gather, manage, and maintain a large qualitative database and to conduct both cross-sectional and longitudinal analyses. Thus, LQR may be overwhelming for novice qualitative researchers. But perhaps more important than knowledge and skills is that researchers who do LQR have relational awareness and are willing to connect with and care about participants [8].

## How does one do LQR?

Like other approaches to qualitative research, the core steps of LQR involve framing research questions, selecting participants, designing data collection instruments (typically interview guides), collecting, organizing and analyzing qualitative data, and drawing novel and useful conclusions. A notable difference in LQR is that the dimensions of time and change affect the complexity of data collection. We tailor-make interviews by asking participants to respond to things they said in past conversations. For example, if someone talked about feeling uncomfortable wearing a white coat as a first-year medical student, we might read back that segment of the interview three years later, and ask, ‘*What comes up for you now as you listen to your former self?*’ In so doing, the participant explicates personal change and guides interpretation of data.

The dimensions of time and change also affect the complexity of data analysis. Data are analyzed cross-sectionally as well as longitudinally. We have found utility in Saldaña’s [2] broad framing, descriptive and interpretive questions. Examples of these questions include what is different from one wave of data collection to another, what becomes more apparent over time, and what is the through-line of this participant’s story, respectively. To analyze individual and group data at a more granular level, we often create data display tables for particularly salient codes or clusters of codes to help us locate patterns in the data. Finally, we have found conceptual frameworks from the social sciences to be a useful lens when looking at time and change, perhaps because LQR challenges the static characteristic of theories commonly applied to health professions education research [7].

## Examples of longitudinal qualitative research

We found it challenging to review the LQR literature because LQR is not always explicitly identified as a specific approach to qualitative research; thus we acknowledge that we may have unintentionally omitted relevant studies. Briefly, seminal LQR studies, such as Thomson and Holland’s investigation of the moral landscape of young people in the United Kingdom [3], are found in the social sciences. In the health sciences, Calman, Brunton, and Molassiotis [9] share lessons learned about LQR and describe a growing number studies that use LQR to explore the experience of patients with chronic illnesses or evaluate long-term outcomes of health-related programs or policies. Within medical education, LQR is relatively uncommon but one area where it has gained a foothold is the exploration of professional identity formation. For example, Babaria [10] in a study of how gendered encounters with patients and supervising physicians impacted medical students’ professional identity formation, interviewed twelve female students over 12 months, after each clinical clerkship. Her analysis was guided by questions such as *‘How is an individual student’s experience similar to, or different from, her previous experience*,’ and ‘*how is this student’s experience similar to or different from the experiences of her peers*?’ In another study of medical students’ professional identity formation, Monrouxe [11] used data from the audio-diaries of 17 students over the first 18 months of medical school. Her analysis was guided by questions such as, ‘*How do physicians-in-training narrate their developing professional identity*?’

We used LQR in a study of differences in the learning experiences of students in two different tracks at the same medical school [12–14]. In the fall of 2010, Columbia University College of Physicians and Surgeons started a new track for a small number of students. The new track featured a longitudinal integrated clerkship at a medical centre in a rural region of the state of New York and had a substantial focus on health systems improvement. Most medical students continued in Columbia’s traditional track, which consisted of rotation-based clerkships in several hospitals in New York City and had limited instruction on health systems improvement. Twenty-three students volunteered to participate in our LQR; nine students in the new track and eleven students in the traditional track stayed with us for a series of five interviews (or five waves of data collection) over the course of four years. Consistently stellar participation rates (85–100% at each wave), in addition to remarkable comments from students like, ‘*Talking to you is one of the few times I reflect on what I’m doing’* and ‘*I gauge my growth as a physician and as a person through these interviews*’, helped us see that our study was not a ‘typical’ qualitative study. Encouraged by students’ sentiments and intrigued by the nature of questions we could explore, we extended our LQR study by following six students in the graduate medical education space. To date, we are in our fourth wave of data collection with these six students-now-residents. The endpoint of our LQR study is yet to be defined.

## Personal reflections on doing longitudinal qualitative research

In reflecting on our LQR study, we compared our experience with LQR to our experience with other research studies. While the core steps of LQR are not unique, two important differences stand out: our shared roles as researchers processing volumes of longitudinal data and dissemination challenges. We have generated guiding principles that we hope will be useful to researchers interested in doing LQR.

### Differences in roles and dissemination challenges

An essential element in the success of our LQR study, to date, has been sharing responsibilities within our working relationship. DB brings technical expertise, passion, and a willingness to engage deeply in the data. She is on the front line of coding and summarizing findings. As an essential complement, BR brings perspective, critical reflection, and vast experience as a generalist in health professions education. In doing LQR, we have learned to jointly explore emergent findings, insights, and questions. DB’s closeness to the transcripts, codes, and themes, allows her to nimbly navigate the database and know what might be found therein. On the other hand, BR’s relative distance from the data allows him to challenge, draw connections, and position findings, insights and questions within the larger field of academic medicine.

Disseminating LQR has been a challenge due to the sheer volume of data, and in our case, the lack of a defined endpoint. We have wrestled to locate cohesive stories within our growing dataset, stories that can be told within the structural format of peer-reviewed journals. Although longitudinal research is often recommended as a next step in health professions education, few actually engage in LQR. In some respects, that gives us an upper hand, so long as we effectively communicate to reviewers that our relatively small sample size, interviewed over the years, has given way to volumes of rich data.

### Our guiding principles for LQR

We believe that guiding principles may help researchers interested in LQR determine both goodness of fit with their research question, and their personal readiness to pursue LQR.

#### Trust

Approach LQR with the primary purpose of building relationships with participants in which they feel safe enough to share details of their experiences. Trust is the ‘fuel’ needed to move initial relationships to long-standing relationships [8]. In our LQR study, for example, we included an incentive when we launched our study but all participants forgot about the incentive after the first year. For them, being heard without judgment, and knowing that we will be there for the long-haul, was incentive enough.

#### Respect

Show respect for LQR participants in multiple ways. If trust is the fuel, respect is the fuel pump. Respect is critical to forging a relationship that will last over the months and the years, and thus minimize attrition in LQR. We show respect for our LQR participants by reading back quotes from their previous interviews. This signifies that their words matter to us. We also show respect for participants’ busy schedules by keeping interviews short (under 60 min) and asking to speak only once or twice a year.

#### Inclusion

Include LQR participants as conversational partners. LQR is typically grounded in philosophical assumptions that align with constructivist paradigms wherein participants are co-constructers of knowledge. Thus, we speak with participants; we do not speak about them. In our LQR study, we routinely ask participants to critique our interpretations and to shape the direction of our inquiry.

#### Tenacity

Capitalize on your intrinsic motivation to do LQR. The bottom line is that LQR requires tenacity. Time is an obvious hurdle, but there is also the analytic burden of cross-sectional analysis, longitudinal analysis, and a comprehensive articulation of the two [2, 7]. LQR is a personal commitment as much as a professional one. For example, we have taken our LQR study with us to new institutions, navigated their institutional review boards, and found ways to incorporate our study into new roles and responsibilities when our ‘day jobs’ have changed.

#### Open-mindedness

Finally, balance tenacity with open-mindedness. Preliminary analysis of each wave of data collection may inform and change the next wave of data collection. We discovered that some questions we pose to the data are applicable cross-sectionally, i. e., to all waves of data collection, while others apply only to longitudinal data. At a more conceptual level, we have learned to stay open to the complex interplay of both individual agency and powerful group norms.

## Conclusion

In summary, we believe that LQR is a useful and coherent approach to qualitative research that allows the exploration of change through time, change in both individual and group trajectories. Although similar to ethnography, participatory action research, and biography in some respects, LQR uniquely focuses on time and change. We offer our reflections and guiding principles to aid researchers interested in doing LQR. As the words of poet Claudia Rankine cited in the preamble suggest [1], LQR is a lesson in patience, but time spent doing LQR opens out to new understanding.
